# Wall slipping behavior of foam with nanoparticle-armored bubbles and its flow resistance factor in cracks

**DOI:** 10.1038/s41598-017-05441-7

**Published:** 2017-07-11

**Authors:** Qichao Lv, Zhaomin Li, Binfei Li, Maen Husein, Dashan Shi, Chao Zhang, Tongke zhou

**Affiliations:** 1 College of Petroleum Engineering, China University of Petroleum, Qingdao, 266580 Shandong China; 2 Chemical and Petroleum Engineering, University of Calgary, Calgary, T2N4V8 Alberta Canada

## Abstract

In this work, wall slipping behavior of foam with nanoparticle-armored bubbles was first studied in a capillary tube and the novel multiphase foam was characterized by a slipping law. A crack model with a cuboid geometry was then used to compare with the foam slipping results from the capillary tube and also to evaluate the flow resistance factor of the foam. The results showed that the slipping friction force *F*_FR_ in the capillary tube significantly increased by addition of modified SiO_2_ nanoparticles, and an appropriate power law exponents by fitting *F*_FR_ vs. Capillary number, *Ca*, was 1/2. The modified nanoparticles at the surface were bridged together and formed a dense particle “armor” surrounding the bubble, and the interconnected structures of the “armor” with strong steric integrity made the surface solid-like, which was in agreement with the slip regime associated with rigid surface. Moreover, as confirmed by 3D microscopy, the roughness of the bubble surface increased with nanoparticle concentration, which in turn increased the slipping friction force. Compared with pure SDBS foam, SDBS/SiO_2_ foam shows excellent stability and high flow resistance in visual crack. The resistance factor of SiO_2_/SDBS foam increased as the wall surface roughness increased in core cracks.

## Introduction

Foam-wall slipping is an important physico-chemical phenomenon, which refers to the sliding motion of foam bubbles on the contact surface of a solid wall. This phenomenon is customarily taken into account when studying foam flow and rheology^1–5^. The study of foam-wall slipping in confined geometries was initially mainly motivated by the successful application of foam in petroleum exploration and production^6, 7^. When foam is injected into formation, it flows through complicated geometries; including cracks, pores, pore throats, etc. Under reservoir conditions, although the friction force created by foam-wall slipping in a micro geometry is very small, a macro-force resulting from the combination of the numerous segments becomes very significant and is critical to a success of production plan.

The foam-wall slipping exhibits a rich behavior depending on bubbles size, gas volume fraction, surface tension, fluid composition, flow rate, etc^8–10^. Experimental and theoretical studies^6, 11–15^ based on different geometries shows that various properties for foam slipping such as friction force and the average film thickness are well fitted by power-low functions with the capillary number, *Ca*,

1 $$Ca=\mu v/{\sigma }$$

where *μ* is the viscosity of continuous phase, *v* is the slipping velocity, and *σ* is the surface tension. Different laws relating friction force and capillary number were identified, with power-law indexes of 1/2, 2/3 and a combination of 1/2 and 2/3. Under 1/2 power law index^11^, foam slipping is characterized by a tangentially immobile surface and a friction dissipation occurring mostly inside the wetting film. Under 2/3 power law index^4^, on the other hand, the slipping is characterized by a tangentially mobile surface and a friction dissipation mainly occurring inside the transition zone between the plateau border and the wetting film. The change between 1/2 and 2/3 power law indexes has been found to closely relate to bubble surface rigidity^13, 16, 17^. On the other hand, using theoretical analysis, Cantat found that for an incompressible (rigid) interface^18^, the friction force exerted by the wall on the liquid meniscus is a combination of two power laws scaled in terms of *Ca* ^1/3^ and *Ca* ^1/2^. Nevertheless, as novel materials and fluid compositions are increasingly employed to produce foam^19–22^, uncertainty still surrounds the study of wall slipping behavior of those new foams, and understanding foam slipping as a basic hydromechanics phenomenon becomes more complex, yet more pressing.

Recently nanoparticle stabilized foams (or Pickering foam) have attracted a considerable interest by virtue of their potential application in enhanced oil recovery, mineral flotation, food industry, cosmetics, ceramics, etc^23–28^. The remarkable feature of the multiphase foams is their stability and foam life that could be extended to weeks or months even under extremely harsh conditions^29–32^. This property benefited from the bubble surface layer formed by carefully selected particles such as silica nanoparticles which are available in well-defined shapes, a spectrum of sizes with narrow size distribution, and the possibility of chemically altering the particle surface^32–34^. The interbubble gas diffusion was prevented and the disproportionation (Ostwald ripening) was slowed down by the resistance of the adsorbed layer of nanoparticles to opening. In addition to stability, foam viscosity was also significantly influenced by the nanoparticles. For example, in a previous work^21^ we have reported a significant increase in the apparent viscosity of a pure surfactant foam with the addition of silica nanoparticles modified by a coating of dimethyl siloxane. Moreover, the nanoparticle-stabilized foam displayed excellent temperature resistance in terms of viscosity. Recent studies contributed to many experimental and theoretical observations pertaining to nanoparticle effect on foam flow. Espinosa *et al*.^35^ reported that for supercritical CO_2_ foam flowed through a capillary, the foam with nanoparticles introduced two to eighteen times increase in the flow resistance factor. The flow resistance factor was defined as the ratio of differential pressure with foam to differential pressure with CO_2_ /brine at same flow rate. Yu and Mo^36^ simultaneously injected CO_2_ and nanosilica dispersion into the porous media of sandstone and then a stable foam was generated. They reported a reduced CO_2_ mobility expressed in terms of the flow resistance factor, which is defined as the ratio of injection pressure of foam to that of CO_2_/ brine at same flow rate. Zheng and Jang reported a two-order of magnitude decrease in the hydraulic conductivity of foam-filled sand columns relative to that of the water-saturated sand column. Sun *et al*.^37^ studied the properties of multi-phase foam and its flow behavior in porous media. They reported an enhancement in the plugging and profile control effects as well as resistance to water flushing in presence of silica nanoparticles compared with pure SDS foam.

Despite the existing literature on nanoparticle stabilized foam, the flow behavior of the foam was majorly based on macro-perspective and the foam was typically studied as a single fluid. Thus the mechanism for the effect of nanoparticle on the foam flow was not clearly identified. More importantly, there are no detailed reports on the wall slipping behavior of nanoparticle stabilized foams. Slipping behavior is critical to the precise evaluation of foam flow for both research and industrial applications. In this paper, we first identify the effect of nanoparticles on foam wall slipping in a capillary tube and model the multiphase fluid flow using a slipping law. The mechanism for the slipping behavior was described from the interaction of the nanoparticles and the surfactant on bubble surface. Then, we compare the foam slipping results from the capillary experiments to a visual crack model employing a cuboid geometry to help understanding the flow resistance of the foam. Finally, we elucidate the effect of crack roughness employing core cracks.

## Materials

Partially hydrophobic SiO_2_ nanoparticles (HDK H15) were purchased from Germany Wacker Chemical, Co., Ltd (Germany). The geometry of the nanoparticles was regular and close to sphere, and the average diameter was approximately 14 nm, per vendor specifications. The surface of the nanoparticles was modified by the vendor with a coating of dimethyl siloxane via covalently bonded silanol group with density of about 1.0/nm^2^, as evaluated by the vendor. The water contact angle on a nanoparticle press cake was about 80°. The volatility at 105 °C was lower than 0.6 wt% for 2 hours and the small weight loss could be neglected at room temperature. Sodium dodecyl benzene sulfonate (SDBS, purity >99.0 wt%, Sigma Aldrich, USA) was used as the foaming agent. Ethanol (purity >99.5 wt%, Sinopharm Chemical Reagent Co., Ltd. China) was used as a co-solvent to solubilize the modified SiO_2_ nanoparticles. Nitrogen (N_2_, purity >99.99 wt%, Tian yuan Inc., China) was used as received. Deionized water was purified by passing through an Elga reverse-osmosis unit and then a milli-Q reagent water system. The surface tension of deionized water measured at 25 °C was approximately 72.0 mN/m. The surfactant-free cleaning agent was made by mixing 67 wt% sulfuric acid (H_2_SO_4_, Sinopharm Chemical Reagent Co., Ltd., China) and 12 wt% of potassium dichromate (K_2_Cr_2_O_7_, Sinopharm Chemical Reagent Co., Ltd. China). Used glassware were cleaned with the agent to avoid the effect of organic contamination. All the measurements were conducted at room temperature (25 °C) unless otherwise specified.

## Methods

### Preparation and characterization of dispersions

In order to prepare well dispersed nanoparticles suspensions, modified SiO_2_ powder was wetted with ethanol first, then mixed with deionized water. The ethanol concentration of the mixture was less than 2.0 wt%. To remove the ethanol, sedimentation-redispersion cycles in pure water was repeated until the residual ethanol was less than 10^−3^ wt%^33^. The SDBS/SiO_2_ dispersions were prepared by adding a known mass of SDBS to the nanoparticle aqueous suspension. The mixture was constantly stirred for at least 10 h and then followed by 20 min of sonication (YP-S17, Hangzhou Success Ultrasonic Equipment Co., Ltd., China) at a frequency of 20 kHz. To avoid overheating, the intervals for work-time and rest-time were set 10 s and 20 s, respectively, and a water bath (F12-EH, Julabo, Germany) was used to maintain the temperature of the dispersion at 25 °C. Finally, the dispersions were sealed for use.

To evaluate the surface properties of bubbles produced with the resultant dispersion, the surface tension and the dilatational viscoelasticity modules were measured through a drop profile analysis interfacial rheometer (Tracker-H, Teclis, France). This technique was successfully used to study rheology of gas–liquid and liquid-liquid interfaces^38–40^. In this work, the same setup was used to evaluate the surface of SiO_2_/SDBS dispersions and nitrogen. To stabilize the surface, droplets from the different dispersions were kept for more than 30 min, then the surface tension was measured by means of axisymmetric bubble shape analysis. Meanwhile measurements of the dilatational viscoelasticity modules were conducted. The droplets were subjected to bubble area sinusoidal oscillations frequency in the range of 0.01 s^−1^–0.1 s^−1^ and the relative amplitude of the bubble area was controlled at 15%. During the measurements, the temperature of the cell was maintained at 25 °C by a water bath.

The viscosity of the dispersions was measured using a rheometer (MCR 302, Anton Paar, Austria) equipped with a concentric cylinder system. A normal cylindrical rotor was used in the measuring system and the shear rate of the rotor was controlled at 100 s^−1^. The temperature of the system was controlled at 25 °C by a semiconductor.

### Foam slipping in capillary tube

**Figure 1:**
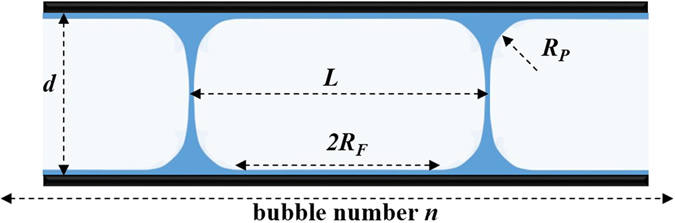
Schematic of foam (bamboo bubbles) in capillary tube (*d* is the diameter of the capillary; *L* is the length of bubble; *R*_F_ is half length of the wetting film and *R*_p_ is the plateau border radius of curvature).

A glass capillary tube with circular cross-section was used to measure the foam slipping behavior. The length of the tube is 30 cm and its diameter is 2 mm. To prepare the bamboo bubbles in Fig. 1, nitrogen was injected into the nanoparticle dispersion, then directly into the tube. The injection velocity, *v*, was controlled by a micro pump (LSP01-2A, Longer, China) with an error of <5 × 10^−3^ mm/s. The gas flow rate and the position of the nozzle with respect to the dispersion surface are important to control the geometry of the foam (bubble organization and size). Once the regular bamboo bubbles were obtained, the excess liquid was removed by gentle rotations and foam drainage to get a desired foam quality. The preparation of the bamboo bubbles took 30 min in order to stabilize the surface property. The volume of the liquid, *V*_L_, in the tube was obtained by weighing the liquid mass, *m*_*L*_, and using a liquid density, *ρ*_*L*_, of 1000 kg/m^3^. The foam quality was calculated by Eq. 2.

2 $${\Gamma }=1-\frac{{V}_{L}}{{V}_{T}}=1-\frac{{m}_{L}/{\rho }_{L}}{nL\pi {d}^{2}/4}$$

where *V*_T_ is the total volume of the foam in the tube and is calculated from the dimensions of the tube as follows *V*_T_ = (*n*−1)*L*π*d* ^2^/4, where *n*, *L* and *d* are given in Fig. 1. The value of the foam quality, *Γ*, was changed from 85% to 98%. Once prepared, the pressure at one end of the tube was recorded by a pressure transmitter (DP1300, Senex, China) and the other end was open to atmosphere. Several cycle operations of forward and backward shifts of foam were used to measure the pressure difference Δ*P* under a constant flow rate *v* in the range 1 mm/s to 30 mm/s. The slipping friction force per spanwise length of bubble surface *F*_FR_^5, 17^ was obtained by the follow expression.

3 $${F}_{FR}=\frac{{F}_{B}}{\pi d}=\frac{{\rm{\Delta }}P\pi {d}^{2}/(4n)}{\pi d}=\frac{{\rm{\Delta }}Pd}{4n}$$

where *F*_B_ is the slipping friction force per bubble and is calculated by Δ*P*π*d* ^2^/(4*n*). The end effects of the tube was neglected given the high number of tested films (*n* ≧ 20). More details about the experiments are provided in the literature^13, 41^.

### Foam flow in cracks

**Figure 2:**
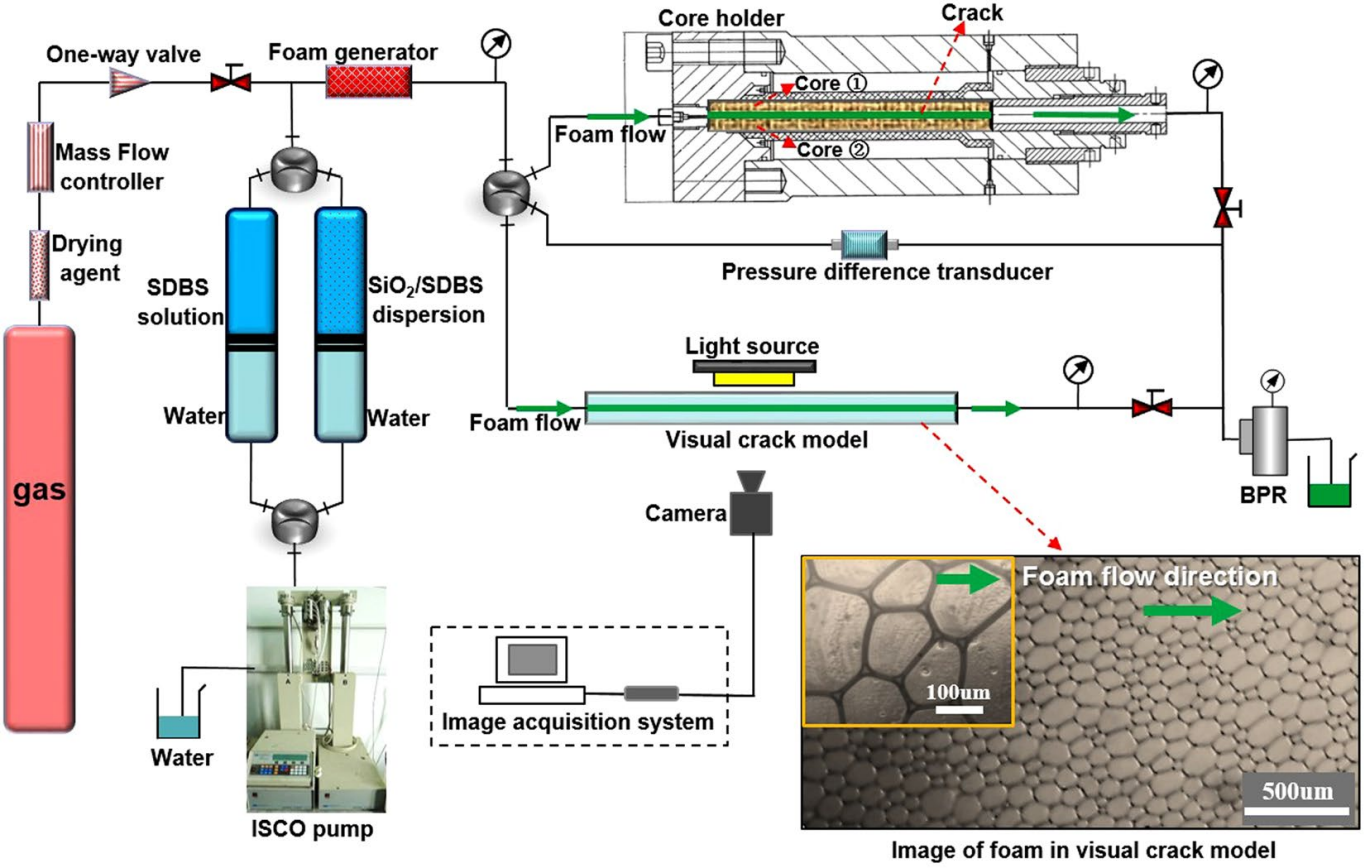
Schematic of foam slipping in core crack and visual crack experimental apparatus.

A laboratory apparatus was designed and built for evaluating the foam flow behavior in cracks. Figure 2 shows a schematic of the apparatus. The setup includes three main parts: a foam generation part, a visual crack model and a core crack model. The foam generation part was used to generate desired nitrogen foams and control the foam flow rate and foam quality. The liquid flow was controlled using an ISCO pump (100DX, ISCO, USA), while the gas flow was adjusted and monitored using a gas flowmeter (F-201CV, Bronkhorst, USA). Spherical ceramsites beads (diameter ≈ 0.4 mm) were used to fill the foam generator (cylindrical with diameter = 6 mm and length = 90 mm) to enable mixing between the nanoparticle dispersion and the nitrogen. The foam quality was varied from 80% to 98% and the foam flow was varied from 0.2 mL/min to 2 mL/min.

#### Foam characterization

After the foam was produced from the foam generator, the foam was transferred to a beaker under nitrogen environment. The bubble surface was scanned using a large depth-of-view portable 3D scanner (VHX-5000, Keyence, Japan) and the roughness of bubble surface was automatically analyzed by focus stacking.

To observe the adsorption behavior of nanoparticles around bubbles, laser scanning confocal microscopy (LSCM, Olympus Fluoview 500, Japan) was refocused on the bubble surface. The modified SiO_2_ nanoparticles were first labeled with fluorescein isothiocyanate (FITC, Sigma Aldrich, USA), and the dispersion was subsequently washed with deionized water to remove the free FITC in the bulk. The fluorescence images were superimposed under blue exciting light.

#### Visual crack model

To study the foam flow behavior in visual cracks, first foam was injected into a horizontal glass crack with a cuboid geometry (gap thickness = 0.1 mm, width = 30 mm, length = 300 mm). The crack was a combination of two glass plates and the gap thickness between the two plates was adjusted by shims. The foam produced by the generator was injected into the crack and flowed through the crack cross-section (0.1 mm × 30 mm). A single layer of bubbles appeared as shown in the illustration of Fig. 2. Measurement of foam flow were taken during steady state after the foam quality was adjusted. A period of 30 min was sufficient to stabilize the foam flow in the crack. The pressure difference between the inlet and the outlet of the crack was recorded by differential pressure sensors. Meanwhile, the microstructure of foam was monitored using a digital microscopic imaging system.

#### Core crack model

Horizontal core cracks with a cuboid geometry (gap thickness ≈0.1 mm, width ≈ 25 mm, length ≈ 100 mm) were used as the flow channel to study the flow behavior of foams. The core crack was made by the combination of two semicylinder cores (core ① and core ② in Fig. 2), which were made by artificial tight sandstone (Bangda Co., Ltd., Hebei, China). The two wall surfaces of the core crack were polished by the manufacturer to get different roughness as detailed in Table 1. To avoid foam leak-off from the long side, the core crack was filled into a core holder and sealed by a rubber sleeve with a confining pressure of 0.5 MPa. It should be noted that the permeability of the cores (<0.1 md) were too small for foam fluid to invade into the matrix under the driving pressure for foam slipping. Thus, the volume of foam filtration was neglected. Before the test, distilled water at 33.3 mm/s was injected into the crack for 5 h to clear the residue. Approximately, 1 h of foam injection was required to stabilize the foam flow in the crack. The pressure difference between the inlet and the outlet of the crack was recorded and the flow rate was controlled in a range from 1.3 mm/s to 13.4 mm/s.

**Table 1:** Properties of core cracks samples.

Core cracks samples	Geometrical parameter			Core matrix Permeability (md)		Mean roughness *R*_a_ of wall surface (μm)	
	Thickness (mm)	Width (mm)	Length (mm)	Core①	Core②	Core①	Core②
#1	0.1	25.1	100.4	0.051	0.062	0.9	1.1
#2	0.1	25.2	100.2	0.079	0.73	3.4	3.1
#3	0.1	25.0	100.1	0.067	0.061	8.7	8.3

## Results and discussion

### Foam slipping behavior

**Figure 3:**
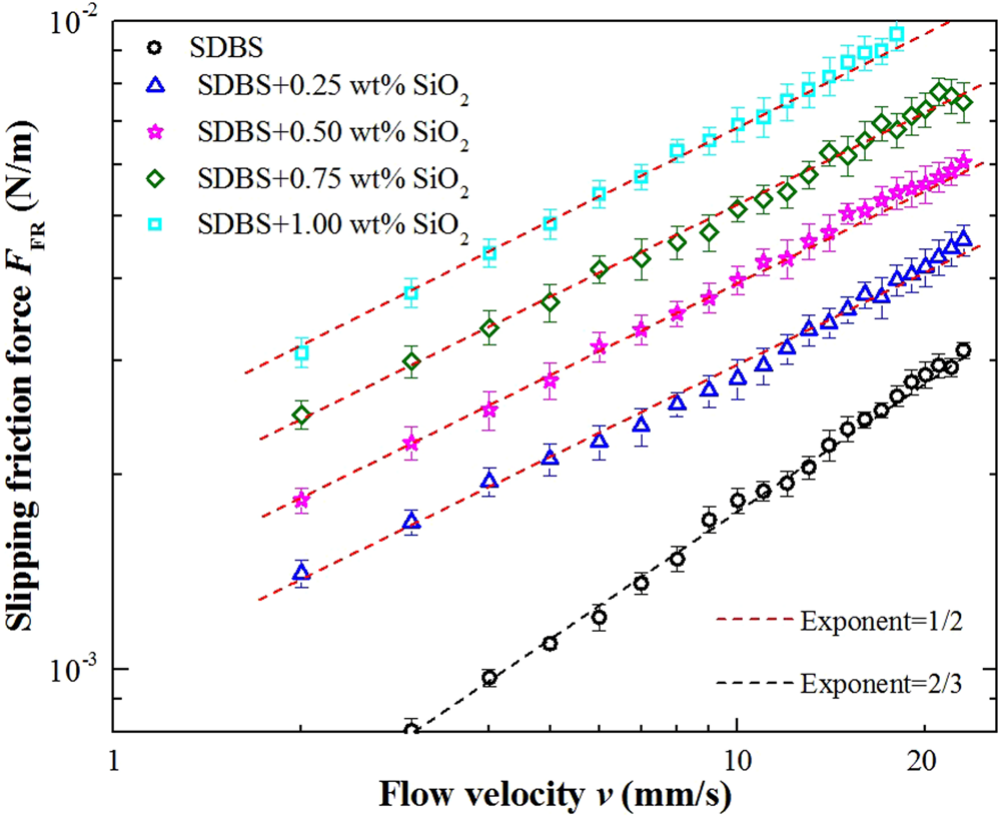
Foam slipping friction force per unit length as a function of flow velocity *ν* for modified SiO_2_ nanoparticle concentration ranging from 0 wt% to 1.00 wt%, fixed SDBS concentration of 1.0 wt% and fixed foam quality of approximately 90%.

**Figure 4:**
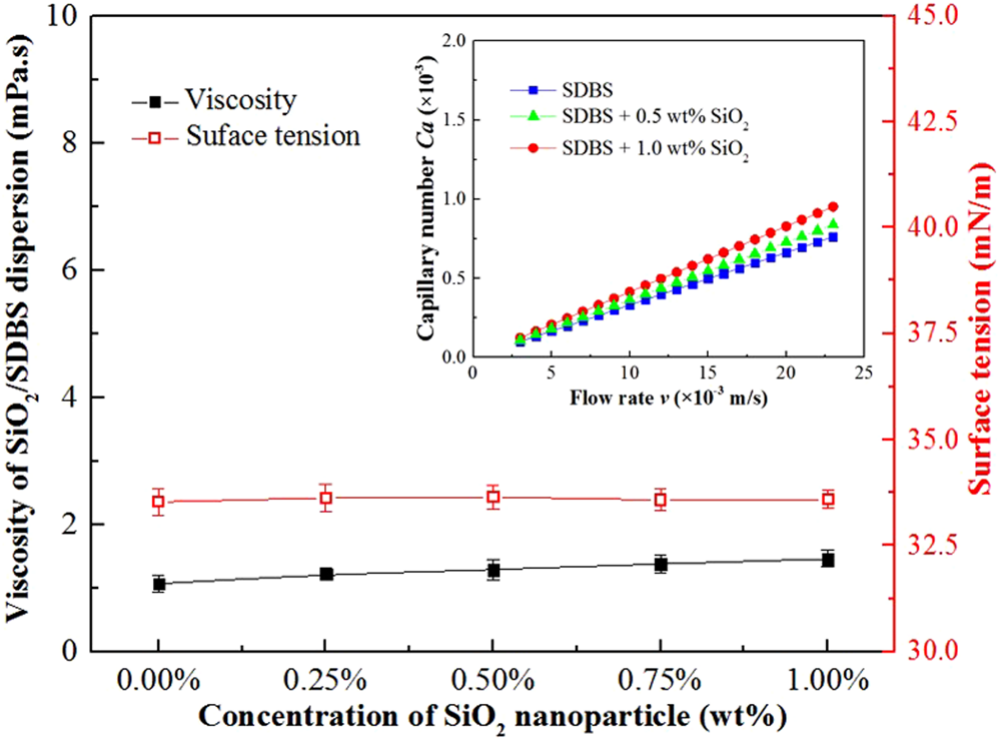
Effect of SiO_2_ nanoparticles on the liquid viscosity *μ*, surface tension *σ* and capillary number *Ca* of SiO_2_/SDBS foam at 25 °C. The concentration of SDBS is 1.0 wt%; the shear rate was 100 s^−1^ during viscosity measurement.

First, the slipping friction force of SiO_2_/SDBS foams in the capillary tube was studied to determine whether the modified silica nanoparticles had any effects on the foam slipping behavior. Figure 3 represents the friction force, *F*_FR_, calculated from Eq. 3, for foams in the absence and presence of different concentrations of the silica nanoparticles vs. flow velocity, *v*. It is obvious that the friction force of silica-containing systems was higher than the pure SDBS foam under the same flow velocity, which suggests that silica nanoparticles increase foam slipping resistance and that there is much more dissipation in SiO_2_/SDBS foams than pure SDBS foams at the contact zones of bubble film with the solid wall of the tube. The viscosity of the continuous liquid phase was one of the determining factors for foam slipping friction force. Figure 4 shows the viscosity of SiO_2_/SDBS dispersion as a function of SiO_2_ nanoparticle concentration. Although the viscosity of the pure SDBS solution slightly increased in presence of the modified SiO_2_ nanoparticles, the value was generally below 1.5 mPa·s. This small change in the viscosity of the continuous phase is not sufficient to explain the significant increase in *F*_FR_. To quantitatively study the effect of SiO_2_ nanoparticles on foam slipping friction force, *F*_FR_ vs. *v* in Fig. 3 was fitted with a straight line. For pure SDBS foams, *F*_FR_ increased as *v* increased from 1 mm/s to 30 mm/s with a power law fit between *F*_FR_ and *v* ^2/3^. This value of exponent was previously attributed to a “mobile” surfactant interface^4^. With the introduction of the modified nanoparticles, *F*_FR_ of the SDBS/SiO_2_ foam was fairly correlated to *v* ^1/2^. Our experimental results showed that the value of the exponent was maintained independent of the number of bubble films. Literature findings suggest that a foam slipping exponent of 1/2 is associated with “immobile” surface^17^. Therefore, we conclude that the slipping regime of the foam changed upon addition of SiO_2_ nanoparticles.

**Figure 5:**
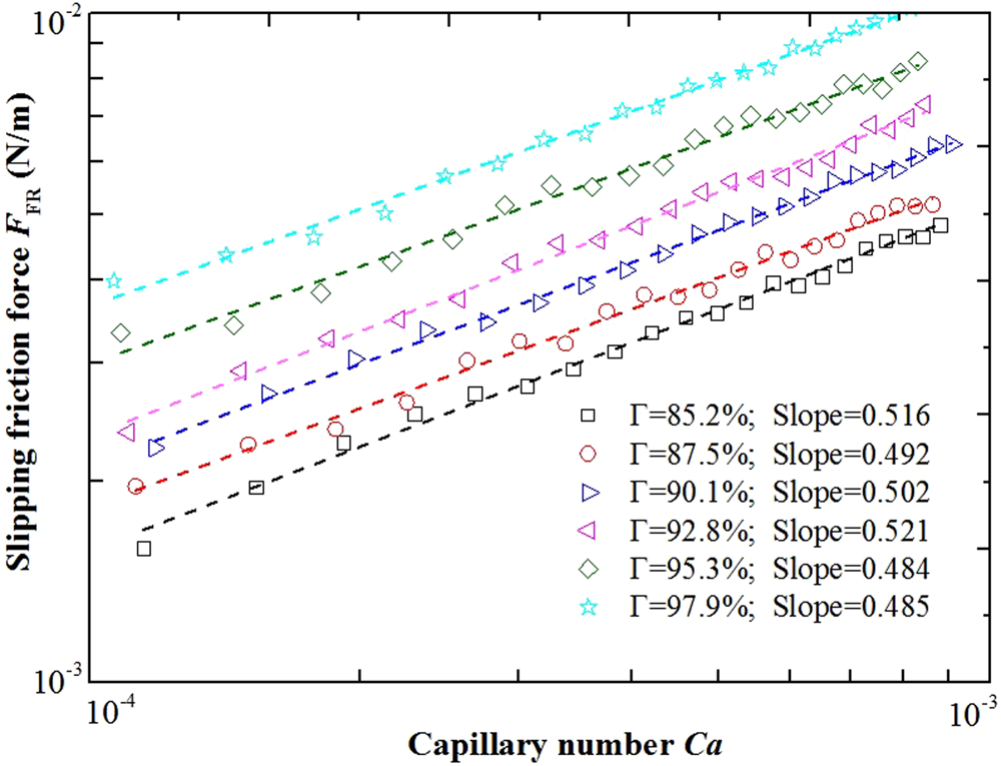
Foam slipping force *F*_*FR*_ as a function of capillary number *Ca* for different foam qualities *Γ*. The slopes of the straight line fit are listed. Concentration of SDBS and silica nanoparticles of the foam were 0.1 wt% and 0.5 wt%, respectively.

Foam slipping models in the literature were mainly power law functions relating *F*_FR_ to the capillary number *Ca* and the foam quality *Γ*. In order to provide a comparison, the SiO_2_/SDBS foam slipping force *F*_FR_ was correlated to the capillary number *Ca* for different foam qualities as shown in Fig. 5. For SiO_2_/SDBS foam with 0.50 wt% nanoparticles and 0.1 wt% SDBS, the slipping force increased when foam quality *Γ* increased from 85% to 98%. A straight line fit of *F*_FR_ vs. *Ca* on the log-log scale displayed a slope of 1/2 ± 4.2% for the different values of *Γ*. Therefore, 1/2 appears to be a suitable power low exponents to fit *F*_FR_ and *Ca*. Again, such a value refelects “rigid” interface during wall slipping measurment^13^. Accordingly, it is concluded that the nanoparticles in fact change the physical character of a foam film leading to a more rigid gas-liquid interface.

For the case of rigid interface, the slipping friction force between bubbles and a solid wall has been derived by Denkov *et al*. based on the lubrication equation^17^. The friction force per unit length of a bubble was given by the following expression.

4 $${F}_{FR}=2.5\sigma {(\frac{Ca{R}_{F}}{{R}_{P}})}^{1/2}+\sigma [7.0C{a}^{3/4}-8.5{(\frac{{R}_{P}}{{R}_{F}})}^{1/4}Ca]$$

where *R*_F_ is the half length of the wetting film and *R*_*p*_ is the plateau border radius of curvature. As shown in Fig. 1, a relationship between the two parameters is given by Eq. 5.

5 $${R}_{F}=(L-2{R}_{P})/2$$

**Figure 6:**
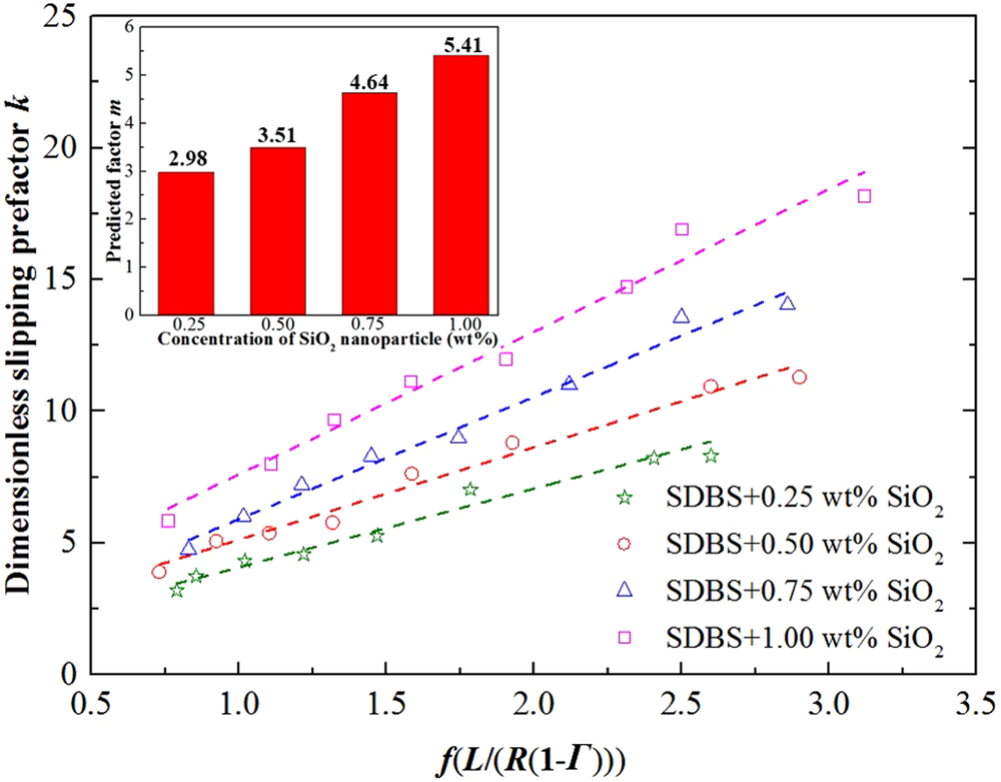
Dimensionless slipping prefactor *k* as a function of *f*(*L/*(*R*(*1* *−* *Γ* ))) = [((4 − π)*L*/(1 − *Γ* )*R*)^1/2^ − 2]^1/2^. The slope of the fitted lines, factor *m*, is presented at different SiO_2_ nanoparticle concentration in the inset. SDBS concentration is 0.1 wt%.

Based on Eq. 4, Emile *et al*.^13^ derived a description of the slipping friction force as a function of the liquid volume fraction, *ε*, and the capillary number, *Ca*. Since the liquid volume fraction *ε* = 1 − *Γ*, we used foam quality *Γ* as a unified parameter defining individual phase fraction. The meniscus radius *R*_p_ = (*dL*(1 − *Γ* )/(8 − 2π))^1/2^, where is the diameter of the capillary tube as shown in Fig. 1. Subsequently, the expression for the friction force was modified to the following.

6 $${F}_{FR}=m{[{(\frac{8-2\pi }{1-{\rm{\Gamma }}}\frac{L}{d})}^{1/2}-2]}^{1/2}\sigma C{a}^{1/2}=k\sigma C{a}^{1/2}$$

where, the dimensionless slipping prefactor *k* is given by Eq. 7,

7 $$k=m{[{(\frac{8-2\pi }{1-{\rm{\Gamma }}}\frac{L}{d})}^{1/2}-2]}^{1/2}$$

whereby *k* is a function of foam quality *Γ* and a geometrical parameter, *L/d*, and *m* is a fitted parameter = 1.77. As shown in Fig. 4, for SiO_2_/SDBS foams, the surface tension *σ* of pure SDBS solution did not change with the addition of SiO_2_ nanoparticles from 0.25 wt% to 1.00 wt%. Likewise, and as indicated above, the viscosity change in the continuous phase was small. Accordingly, at a constant flow rate *v*, the capillary number *Ca* of SDBS foam did not change much by the addition of SiO_2_ nanoparticles. However, as shown in Fig. 3, the friction force of SiO_2_/SDBS foam is strongly correlated to the nanoparticle concentration. In order to quantitively analyze the nanoparticle effect on *F*_*FR*_, the slipping prefactor *k* was calculated from Eq. 6 for given values of *F*_*FR*_, *σ* and *Ca* at different SiO_2_ nanoparticle concentration and plotted vs. *m*[((8 − 2π)*L*/((1 − *Γ* )*d*))^1/2^ − 2]^1/2^ from Eq. 7 in Fig. 6. According to Emile model in Eq. 6, the dependence should be linear with a slope *m* = 1.77. However, for SiO_2_/SDBS foams, the value of *m* in Fig. 6 is higher than 1.77 and increased monotonically with nanoparticle concentration from 0.25 wt% to 1.00 wt%. Thus, the slipping model for SiO_2_/SDBS foam with nanoparticle should be purely phenomenological. This increase in factor *m* with nanoparticle concentration suggests much higher dissipation is experienced in presence of the nanoparticles. The formula to describe the relationship between *m* and nanoparticle concentration could be easily obtained by experimental data fitting. However, for the prediction of foam friction force, it is meaningless to build an empirical model of *F*_*FR*_ as a function of nanoparticle concentration, because the foam property is not only decided by the nanoparticle concentration, but also depends on the surfactant as well as the interaction between the surfactant and the surface of the particles. Subsequently, effort was directed to understanding the mechanism of foam slipping behavior through analyzing the interactions between the nanoparticles and the surfactant on the bubble surface.

### Bubble surface property

**Figure 7:**
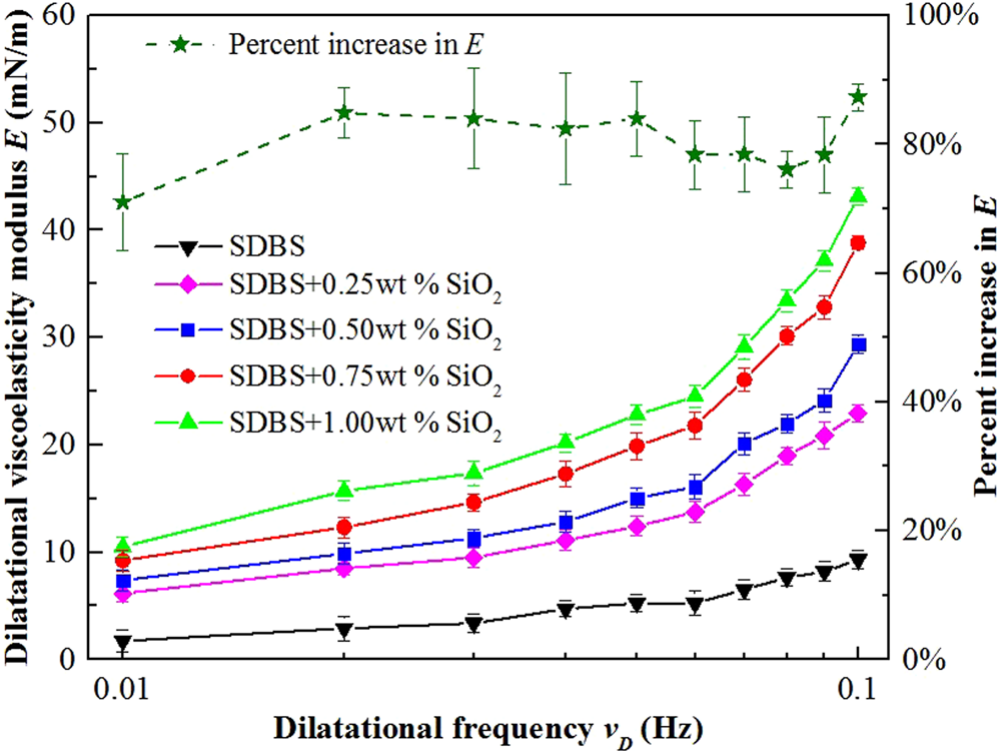
Dilatational viscoelastic modulus *E* of bubble surface as a function of dilatational frequency *v*_*D*_. SDBS concentration was 0.1 wt %. The percent increase in *E* between the lowest, 0.25 wt% SiO_2_, and the highest 1.00 wt%, SiO_2_, nanoparticle concentration calculated from Eq. 8 is plotted on the secondary y-axis.

In order to draw insightful conclusion on the slipping mechanism, the bubble surface properties, which govern the slipping behavior, were studied from micromechanics and microstructures aspects. The slipping force is a function of capillary number *Ca* which contains a parameter *σ*, and the surface tension *σ* is one of critical parameters to describe bubble surface property. However, as shown in Fig. 4, for foam with 0.1 wt% SDBS surfactant, the value of surface tension was not significantly affected by the change in nanoparticle concentration. Nevertheless, when considering the gradient of the surface tension, measured as the dilatational surface viscoelastic modulus, the effect of nanoparticle became clearer. The dilatational viscoelastic modulus, *E*, of bubble surface in the absence and presence of SiO_2_ nanoparticles with concentrations varying from 0.25 wt% to 1.00 wt% was studied. The value of *E* measured by interfacial rheometer as a function of oscillation frequency from 0.01 s^−1^ to 0.1 s^−1^ is plotted in Fig. 7. The modulus *E* increased as nanoparticle concentration increased. The viscoelasticity modulus represents mechanical strength of the surface and reflects the resistance toward interfacial perturbation or deformation^42^. The value of *E* increased with SiO_2_ nanoparticle concentration revealing higher resistance to deformation, which suggests more rigid bubble surface. This result is in agreement with the SDBS/SiO_2_ foam slipping exponent of 1/2 reported above.

**Figure 8:**
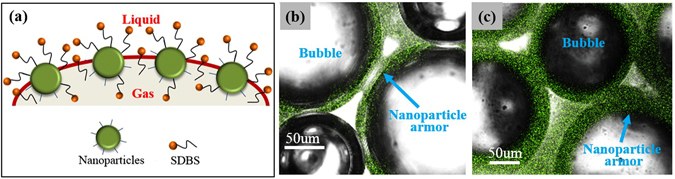
(**a**) Illustration of nanoparticle-surfactant interaction and nanoparticle adsorption on bubble surface. Confocal fluorescence image of SiO_2_/SDBS foams: (**b**) 0.25 wt% SiO_2_ nanoparticle + 0.1 wt% SDBS + N_2_; (**c**) 1.00 wt% SiO_2_ nanoparticle + 0.1 wt% SDBS + N_2_.

The effect of SiO_2_ nanoparticles on the surface could be explained by considering nanoparticle-surfactant interactions. As schematically shown in Fig. 8(a), it is highly likely that the SDBS surfactant is adsorbed on the surface of the modified silica nanoparticles through hydrophobic interactions, while its hydrophilic head group faces outwards. This configuration has been verified by zeta potential measurements from our previous work^43^. Consequently, SDBS adsorption confers surfactant-like properties onto the SiO_2_ nanoparticles, which increase their partitioning onto the gas–water interface. In order to verify the adsorption of nanoparticle on the bubble surface, we focused LSCM on the SDBS/SiO_2_ bubble. As shown in Fig. 8(b) and (c), the adsorption of SiO_2_ nanoparticles on the bubble surface could be clearly observed. It appears that the SiO_2_ nanoparticles at the surface were bridged together and formed a dense particle “armor” around the bubble. The interconnected structures of the “armor” with strong steric integrity contributed to solid-like surface. Thus, as indicated above, nanoparticle armored bubble showed a slipping behavior similar to previously studied rigid surfaces.

Moreover, confocal fluorescence images in Fig. 8(b) and (c) showed that when nanoparticle concentration increased from 0.25 wt% to 1.00 wt%, the nanoparticle adsorption behavior became more evident and the thickness of the nanoparticle armor increased. Thus, the solid-like behavior of the surface became more pronounced and higher mechanical strength is attained with the increase in modulus *E* with the increase in nanoparticle concentration as shown in Fig. 7. To quantitatively compare the results of this surface measurement with related foam slipping measurement, the changes of surface modulus and foam slipping force were analyzed based on their percent increase. The percent increase in *E* with nanoparticle concentration calculated from Eq. 8 below was also included in Fig. 7.

8 $${\rm{Percent}}\,{\rm{increase}}\,{\rm{in}}\,E=\frac{{E}_{1.00wt \% {{\rm{SiO}}}_{{\rm{2}}}}-{E}_{0.25wt \% {{\rm{SiO}}}_{{\rm{2}}}}}{{E}_{0.25wt \% {{\rm{SiO}}}_{{\rm{2}}}}}$$

As the dilatational frequency increased from 0.01 s^−1^ to 0.1 s^−1^, the percent increase in *E* fluctuated between 73% and 90%. For the slipping friction force of the SDBS/SiO_2_ foam, *m* varied from 2.98 to 5.41 with nanoparticle concentration, as shown in the inset of Fig. 6. Therefore, *m* increased by 82% as *E* changed approximately with a same increased ratio. This result was compared with a previous foam slipping study for surfactant mixture systems. Emile *et al*.^13^ reported an exponent of 1/2 to describe foam slipping with solutions where high dilatational modulus were obtained. However, in their study the slip factor *m* only varied by 30% (82% in this study) as *E* varied by 2 orders of magnitude (only increased by 73% to 90% in this study). The difference of the experiment data between the two studies is too large to be neglected. Moreover, the theoretical model describing the friction force on bubble with rigid interface (with high surface viscoelastic modulus *E*) developed by Cantat^18^ was fit to the data in Figs 3 and 5, but produced large error. Consequently, for foams with nanoparticle-armored bubbles, a single value of *E* is informative but insufficient to explain the change of slipping friction force of foam with nanoparticles. Accordingly, more analysis was deemed important.

**Figure 9:**
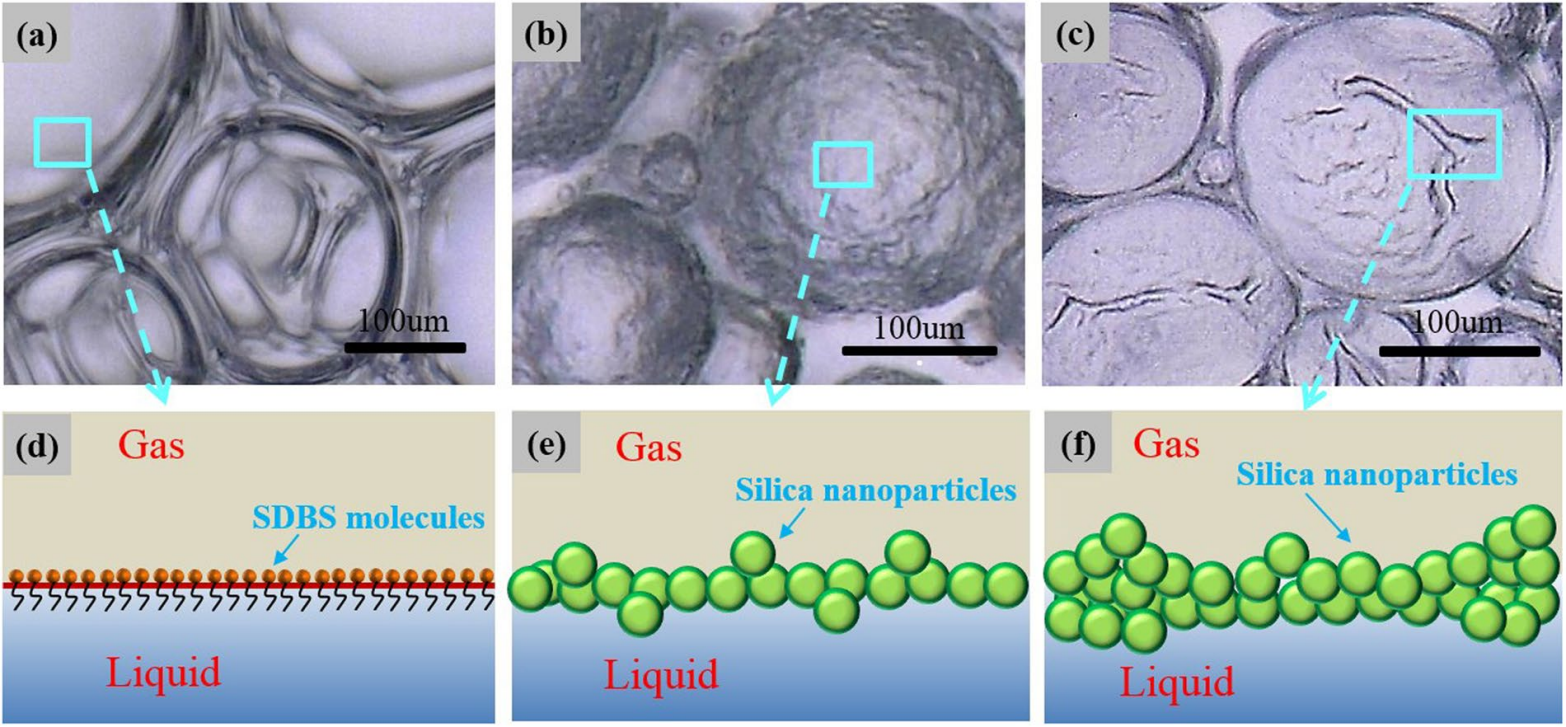
3D microscopy images and the corresponding schematic illustrations of the microscopic structure of the bubble surfaces of (**a**,**d**) SDBS foam: 0.1 wt% SDBS + N_2_ **;** (**b**,**e**) SiO_2_/SDBS foam: 0.25 wt% SiO_2_ nanoparticle + 0.1 wt% SDBS + N_2_ **;** (**c**,**f**) SiO_2_/SDBS foam: 1.00 wt% SiO_2_ nanoparticle + 0.1 wt% SDBS + N_2_.

In addition to evaluating micromechanics of bubble surface, the microstructure of the armored bubble was studied. The bubble surface was scanned and the 3D microscopic images is shown in Fig. 9. For pure SDBS foam (Fig. 9(a)), the bubble surface was very smooth, and as schematically illustrated in Fig. 9(d), the surface was occupied by free SDBS surfactant molecules and the layer was fluid-like. As the surface tension was uniformly distributed on the surface layer, the bubble surface was stretched and showed a smooth microstructure. For SiO_2_/SDBS foam with 0.25 wt% nanoparticles, the bubble surface appeared rough as in Fig. 9(b). This was mainly due to irregular compression of the nanoparticles on the surface, as illustrated in Fig. 9(e). Compared to surfactant adsorption, the adsorption of the nanoparticles was irreversible and stable. Thus, further compression caused by the layer tended to be undulated and the morphology was very stable. When the nanoparticle concentration of the SiO_2_/SDBS foam increased to 1.00 wt%, Fig. 9(c) depicts there were out-of- plane distortions at random positions. The bubble surface became crumpled and appeared much rougher. As illustrated in Fig. 9(d), much more nanoparticles adsorbed on the surface irregularly and caused the layer to be more solid-like. Thus, the mechanical intensity of the nanoparticle adsorbed layer was high enough to overcome the surface tension, and the distortion of the surface could not be stretched to a curvature morphology by the surface tension. The roughness of the bubble surface increased with nanoparticle concentration, which in turn changes the slipping friction force. Rough surfaces can cause a wetting film to become narrower in some part, which increases the velocity gradient in the film leading to a major increase in viscous forces. Moreover, some rough surfaces with solid-like property may touch the solid wall causing solid to solid friction, which increases the resistance coefficient in the contact zones. Subsequently, the slipping friction force could be easily increased.

### Foam flow in cracks

The potential application of foam in the oil and gas industry has created an immediate demand for foams with high performance^44–46^. Foams flow resistance in cracks are widely required for reducing loss of fracture fluids, profile control, water shutoff, drilling fluid filtration control, etc. Foams flow resistance in cracks results from combining the flow resistance of each bubble, which closely relates to its wall slipping friction force. In this part, a crack model with a cuboid geometry was used to further study foam flow behavior, which was compared with the results from foam slipping in capillary. The results were used to evaluate the foam flow resistance enhancement by nanoparticles.

#### Visual crack flow

**Figure 10:**
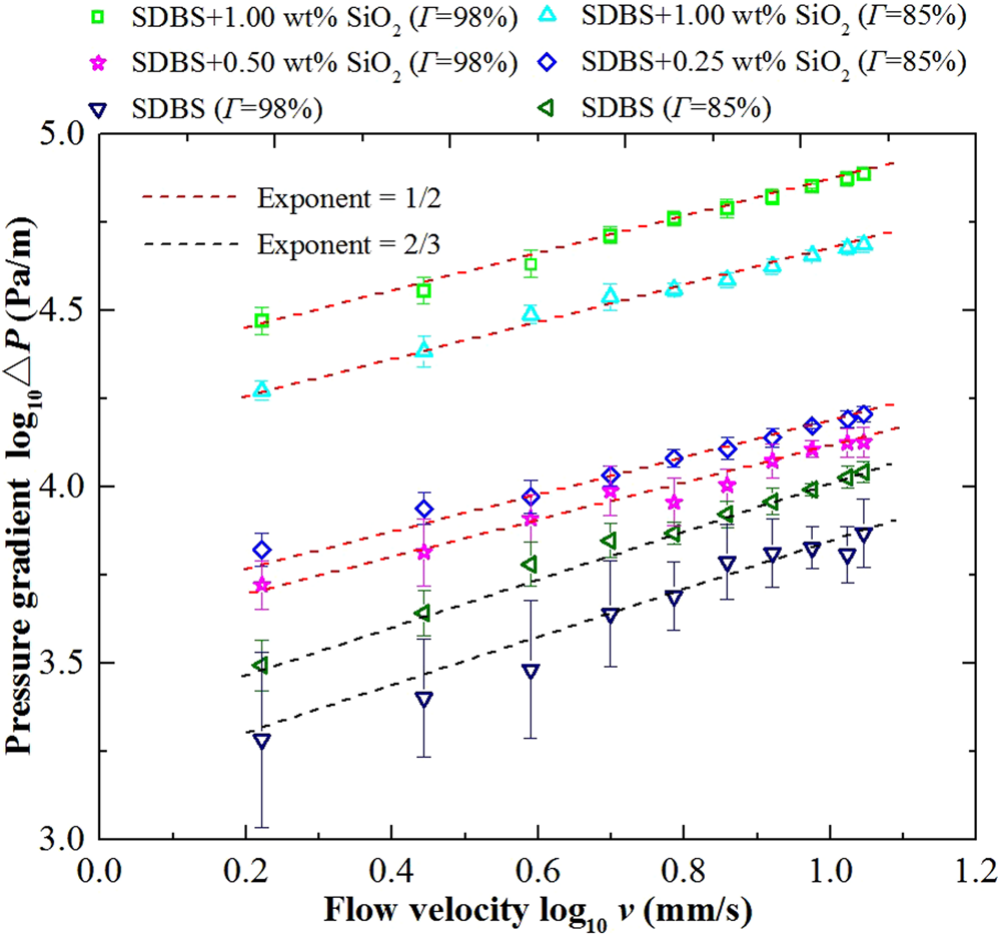
Logarithm of pressure gradient ∆*P* for foam flow in a crack vs logarithm of flow velocity *v* at two foam qualities: 85% and 95%. SDBS concentration was kept at 0.1 wt% and SiO_2_ nanoparticle concentration was varied from 0.25 wt% to 1.00 wt%.

First, the pressure gradient during foam flow in the visual crack was measured as a function of flow velocity for foams with different nanoparticle concentrations and different qualities. As shown in Fig. 10, each foam displays the same general trend of increasing pressure gradient with increasing flow velocity. For SDBS/SiO_2_ foams and SDBS foams, the pressure gradient and velocity could be fitted by power law with 1/2 and 2/3 exponents, respectively, which is in agreement with the law of foam slipping force established in capillary tube. Fits with 1/2 exponent suggest that the friction dissipation of SDBS/SiO_2_ foams in the crack also occurs mostly inside the wetting film, in a similar fashion to a capillary. Nevertheless, when the effect of foam quality was considered, the crack experiments displayed different results from their capillary counterparts. In a capillary, as shown in Fig. 5, the foam slipping force increased with increasing foam quality, whereas in a crack, for SDBS/SiO_2_ foams with 0.25 wt% silica nanoparticle, the pressure gradient measured at foam quality of 85% was higher than that measured at foam quality of 98%. The same observation was reported during SDBS foam flow in the crack for the above foam qualities. Whereas for foams with higher nanoparticle concentration, i.e. 1.00 wt%, an opposite trend in the pressure gradient was observed.

**Figure 11:**
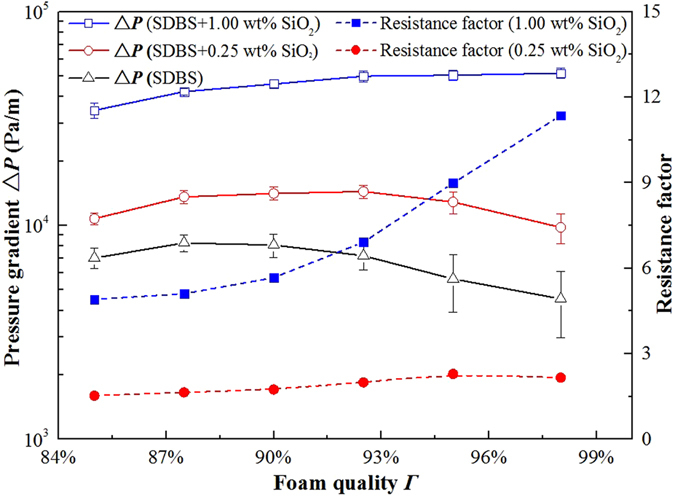
Pressure gradient ∆*P* and foam resistance factor as a function foam quality *Γ*. SDBS concentration was kept at 0.1 wt% and SiO_2_ nanoparticle concentration was 0.25 wt% or 1.00 wt%. Flow velocity ν was kept at 5 mm/s.

**Figure 12:**
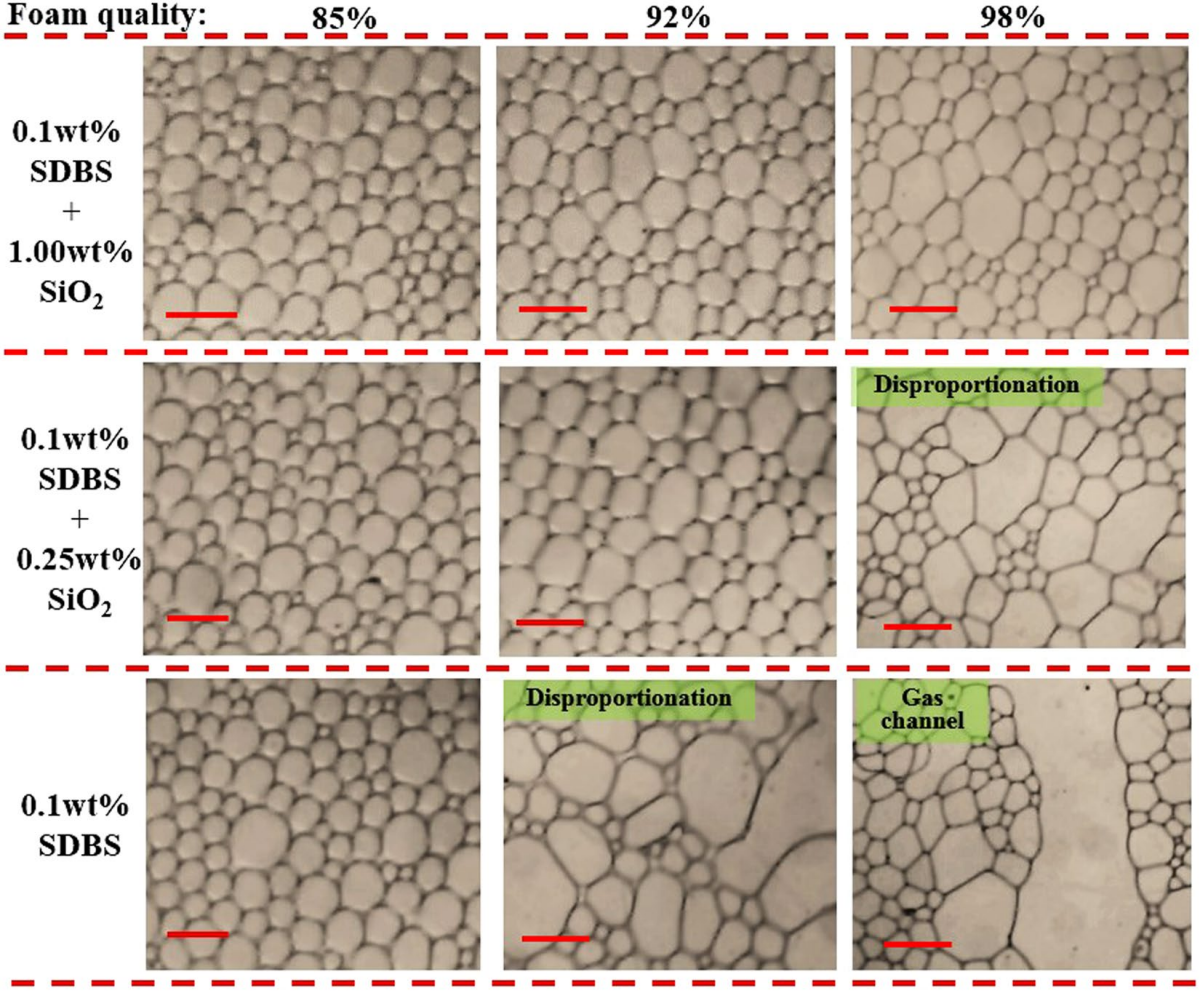
Optical micrographs showing different foam microstructures during flow in cracks for different SDBS/SiO_2_ foam quality and nanoparticle concentration. SDBS concentration was 0.1 wt% and flow velocity *ν* was kept at 5 mm/s. The rad scale bar is 300 μm.

To further study the effect of foam quality during foam flow in cracks, the pressure gradient Δ*P* was measured as function of foam quality *Γ*. The results are plotted in Fig. 11. Meanwhile, the foam microstructure during flow in the crack was monitored and the micrographs at foam qualities of 85%, 92% and 98% are included in Fig. 12. For pure SDBS foam, the pressure gradient first increased slightly to a maximum, and then decreased with increasing foam quality. This result could be explained by considering the microstructure of foam. As foam quality increased from 85% to 98%, Fig. 12 shows that the film in each foam system became thinner. For pure SDBS, high quality foams displayed very fragile and sensitive films to disturbance such as interactions between gas bubbles and pressure fluctuation. Thus, film rupture occurred easily and bubble disproportionation of SDBS foam was observed clearly at foam quality 92%, as depicted in Fig. 12. With foam quality increasing to 98%, the foam was very unstable and gas channels formed leading to low foam flow resistance. For a foam with 0.25 wt% SiO_2_ nanoparticles, bubble disproportionation also occurred at high foam quality, *Γ* = 98%, meanwhile the pressure gradient of foam became lower. With the addition of SiO_2_ nanoparticles, with concentration increasing to 1.00 wt%, sufficient nanoparticles adsorbed at the gas–liquid interface forming a dense layer, thus slowing down or completely halting disproportionation. The films were very stable from quality between 85% and 98%. In this case, the pressure gradient increased with increasing foam quality even at high foam quality. To evaluate the enhancement of foam flow resistance by the addition of nanoparticles, the resistance factor was calculated from pressure gradient ratio as given in Eq. 9.

9 $${\rm{Resistance}}\,{\rm{factor}}=\frac{{\rm{\Delta }}{P}_{{\rm{foam}}{\rm{with}}{\rm{nanoparticle}}}}{{\rm{\Delta }}{P}_{{\rm{pure}}{\rm{surfactant}}{\rm{foam}}}}$$

As shown in Fig. 11, with the addition SiO_2_ nanoparticles, resistance factors belonging to 0.25 wt% and 1.00 wt% nanoparticles increased with foam quality. Especially for foam with 1.00 wt% SiO_2_ nanoparticles, the resistance factor displayed a pronounced increase at high foam quality range, 90% to 98%, which suggests that the flow resistance of foam was significantly improved by the addition of nanoparticles.

#### Core crack flow

**Figure 13:**
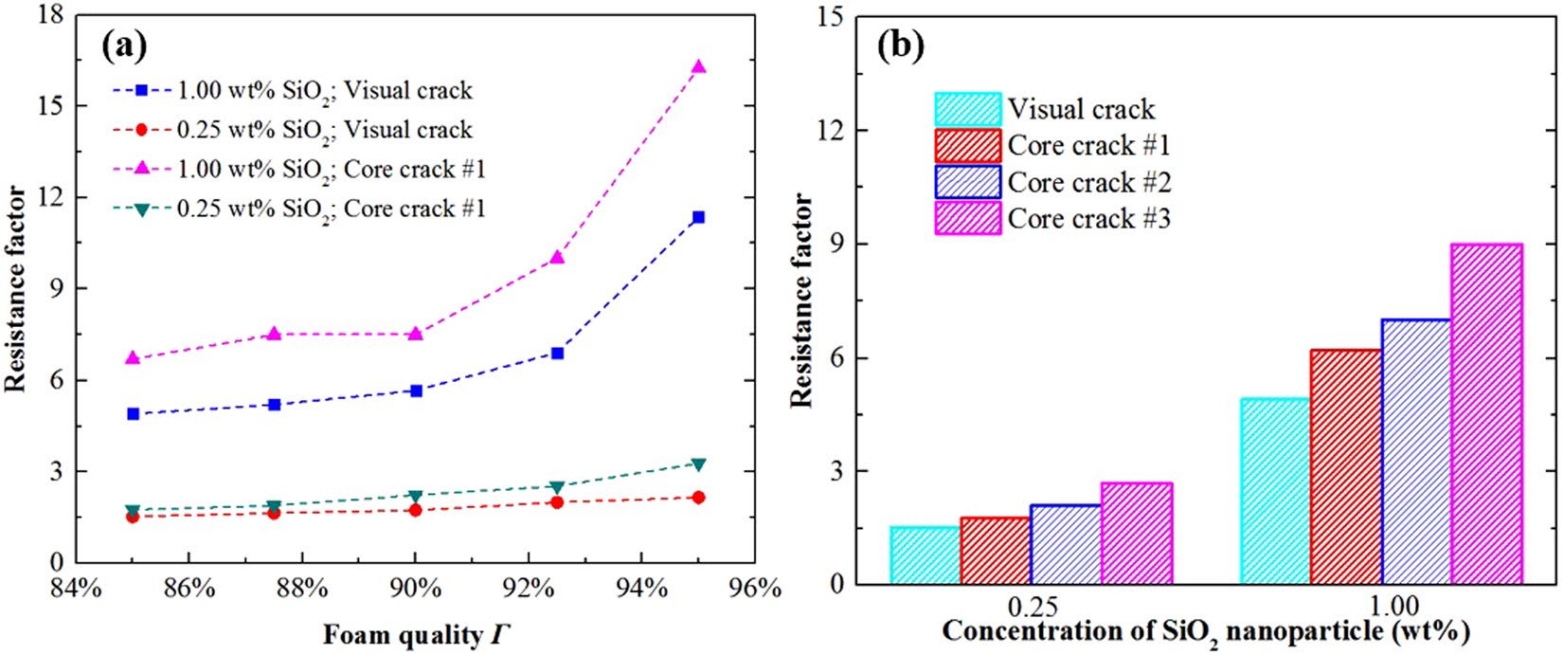
(**a**) Foam resistance factor in visual and core cracks vs foam quality. (**b**) Foam resistance factors for visual and core cracks with different surface roughness. Foam quality 85%, surface roughness of core cracks increased from 1 to 3 and is included in Table 1. SDBS concentration was kept at 0.1 wt%, foam flow velocity ν in cracks was kept at 5 mm/s.

For oil and gas development applications, foams are typically directly injected into formation. Hence, the purpose of the core crack experiments was to closely model foam flow in a formation crack. First, the flow resistance enhancement in the core crack was evaluated and the resistance factor of foams was compared with that in the visual crack. As shown in Fig. 13(a), when the pressure drop for SDBS/SiO_2_ foams were measured in core crack, a much higher value of resistance factor was obtained, which indicates a high foam slipping friction force and a much more effective foam flow resistance occurred in core cracks. The biggest difference between the visual crack and the core cracks was that the wall surface of the former is smooth, while that of the latter is rough. As stated earlier, the roughness of the bubble surface increased with the addition of nanoparticles. Subsequently, it is intuitive to conclude that the contact of the two rough surfaces should be responsible for changing the foam flow behavior. To verify this conjecture, core cracks with different wall roughness were used to measure the resistance factor of the SDBS/SiO_2_ foams. Figure 13(b) compares the resistance factor *b* for two different nanoparticle concentrations for surfaces with different roughness. The resistance factor increased with surface roughness and the increase was more pronounced for the foam with 1.00 wt% nanoparticles, since the surface of nanoparticle armored bubbles tend to be more solid-like and rougher at higher nanoparticle concentration. According to the conventional mechanical friction theory, the more rough and jagged the surface is, the more easily its molecules to contact the molecules of the surface it touches. Consequently, higher friction force is exerted and foam resistance factor in cracks increased.

**Figure 14:**
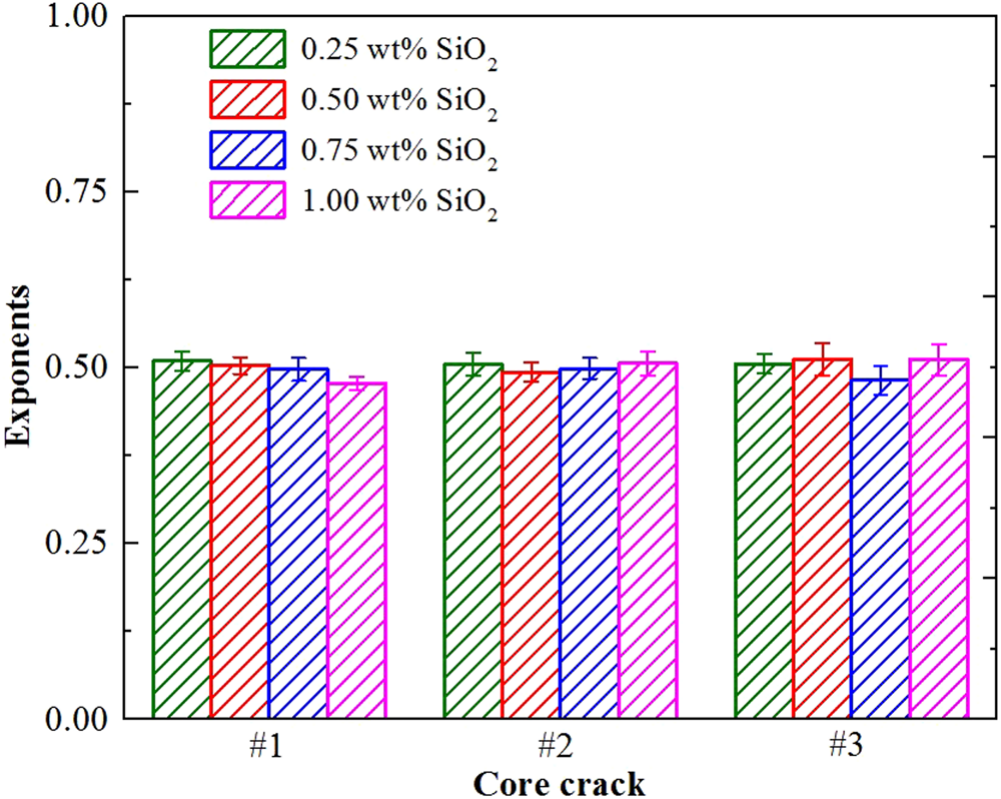
Exponents of power law fitting for the pressure gradient of SDBS/SiO_2_ foam vs flow velocity ν in different core cracks. SDBS concentration was kept at 0.1 wt%.

To study foam flow regime in the different core cracks, the pressure gradient as a function of velocity was fitted by power law and the exponents of that fit are included in Fig. 14. A power law exponent around 1/2 was obtained for the different nanoparticle concentration, independent of the crack wall surface roughness. This suggests that, while the flow behavior of SiO_2_/SDBS foam was significantly influenced by the wall surface roughness, the flow regime did not changed in the core crack models and dissipation is dominated by the wetting film. Finally, according to foam flow experiments in cracks and surface measurements, surface roughness and foam stability was enhanced by the nanoparticle armor leading to a solid-like surface, which impacted foam flow resistance with minimum effect on flow regime.

## Conclusion

In this paper, wall slipping behavior of foam with nanoparticle armored bubbles was systematically studied and its flow resistance was evaluated in visual and core cracks having different surface roughness. The following conclusions can be drawn from this work.

1. The results of foam slipping in capillary tube indicated that slipping friction force, *F*_FR_, significantly increased by addition of the partially hydrophobically modified SiO_2_ nanoparticles. The slight change in the viscosity of the foam continuous phase was not sufficient to explain the increase in the foam slipping friction force. The slipping force *F*_FR_ increased when foam quality *Γ* increased from 85% to 98% and in this range, a power law with 1/2 exponent fitted the *F*_FR_ and *Ca* data to a good extent.
2. Micrographs confirmed that the modified nanoparticles absorbing at the surface were bridged together and formed a dense particle “armor” around the bubble. The interconnected structures of the “armor” with strong steric integrity confer a surface solid-like, and the dilatational surface viscoelastic modulus *E* of the foam increased. The result was in agreement with the slipping exponent 1/2 of SDBS/SiO_2_ foam which was associated with rigid surface.
3. A single value of dilatational surface viscoelastic modulus *E* was informative, however not sufficient to explain the significant change in the slipping friction force. The roughness of the bubble surface increased with nanoparticle concentration, which provided a more reliable explanation for the increase in the slipping friction force.
4. Compared to pure SDBS foam, SDBS/SiO_2_ foam show an excellent stability and a high flow resistance in a visual crack, especially within the high foam quality range. For foam with 1.00 wt% SiO_2_ nanoparticles, the increase in the resistance factor with foam quality was the most significant.
5. In core cracks, the resistance factor of SiO_2_/SDBS foam increased with the surface roughness without impacting the flow regime and dissipation was dominated by the wetting film.
